# Post-traumatic Cervical Osteomyelitis Masquerading as Neck Strain Following Minor Trauma: A Case Report

**DOI:** 10.7759/cureus.89117

**Published:** 2025-07-31

**Authors:** Chinelo G Meniru, Sheri P Walls, Nkechi Ukoha, Adedoyin Ademiluyi

**Affiliations:** 1 Internal Medicine, Piedmont Athens Regional Medical Center, Athens, USA; 2 Internal Medicine, Universidad Iberoamericana, Mexico City, MEX

**Keywords:** cervical osteomyelitis, cervical spinal cord compression, delayed diagnosis, discitis, multidrug antibiotic therapy, musculoskeletal pain, neck strain, osteomyelitis, spinal infections

## Abstract

Osteomyelitis is an infection of the bone tissue, causing inflammation. Common causes and risk factors include trauma, surgical procedures, the presence of foreign bodies, and immunocompromised states such as Human Immunodeficiency Virus (HIV) infection and diabetes mellitus. Osteomyelitis occurring in the absence of risk factors is relatively rare.

Herein, we present a case of a 41-year-old female dog groomer with a history of iron deficiency anemia and no other significant chronic medical conditions, who presented with persistent neck pain and bilateral hand numbness following a work-related incident in which a dog she was grooming pulled her forward, causing a popping sensation in her neck. Imaging revealed destructive changes at C6-C7, concerning for osteomyelitis, with resulting cervical kyphosis and severe spinal cord compression. Intravenous (IV) antibiotics were started, and she underwent neurosurgical intervention, which revealed a phlegmon and small abscess intraoperatively. Although bone biopsy and blood cultures were negative, likely due to prior initiation of antibiotics, empiric antibiotic therapy was continued for six weeks based on clinical suspicion and surgical findings.

This case highlights the importance of maintaining a broad differential when evaluating persistent neck pain, particularly after minor trauma. Increased awareness of these atypical presentations may facilitate earlier diagnosis and improve outcomes in otherwise low-risk individuals.

## Introduction

Osteomyelitis is an infection of bone tissue that leads to progressive inflammation and, if untreated, destruction of the bone and surrounding structures. It can occur through various mechanisms, including hematogenous spread, contiguous spread from adjacent soft tissue infection, or direct inoculation from trauma or surgical procedures [1]. Many contributing factors predispose a patient to developing osteomyelitis, including age, diabetes, peripheral vascular disease, IV drug use, surgical implants, and immunodeficiency due to disease or immunosuppressant drugs [1].

Osteomyelitis in the cervical spine is uncommon, representing a small fraction of all vertebral osteomyelitis cases, which themselves are relatively rare [2]. When it does occur, it is often associated with significant morbidity due to the proximity of neurovascular structures and the risk of spinal cord compromise [2]. The condition is particularly unusual in patients without traditional risk factors, making diagnosis more challenging and delays more likely.

Clinical presentation can vary depending on the site and acuity of the infection. Symptoms often include localized pain, swelling, warmth, and sometimes systemic signs like fever or malaise. Some mimics of cervical osteomyelitis include arthritis, such as rheumatoid arthritis, metastatic bone disease, and fractures, including pathological and stress fractures [3].

Diagnosis typically requires a combination of clinical evaluation, laboratory tests, microbiological cultures, and imaging studies. While plain radiographs may be used as an initial modality to exclude other pathologies or detect chronic changes, magnetic resonance imaging (MRI) is the most sensitive and most specific imaging modality for the detection of osteomyelitis and provides superb anatomic detail and more accurate information about the extent of the infectious process and soft tissues involved [3]. Bone biopsy (either open or percutaneously) is essential to establish the histopathological diagnosis in osteomyelitis, identify the causative pathogen, and provide susceptibility data that helps direct antibiotic therapy. Nuclear imaging has a high sensitivity for detecting early evidence of bone disease, but has very poor specificity. It is especially useful if metal hardware prevents the use of an MRI. Three-phase technetium-99 bone scan and tagged white blood cell scans are the modalities commonly used [3].

In this report, we present the case of a previously healthy middle-aged woman with no known comorbidities who developed cervical osteomyelitis following a work-related neck injury initially presumed to be mechanical in nature. This case highlights the importance of maintaining a broad differential diagnosis when evaluating persistent neck pain, particularly after minor trauma, even in the absence of systemic illness or classical predisposing conditions. Increased awareness of these atypical presentations may facilitate earlier diagnosis and improve outcomes in otherwise low-risk individuals.

## Case presentation

This is a case of a 41-year-old female dog groomer, with a history of iron deficiency anemia and no other chronic medical conditions, who presented with progressively worsening neck pain and bilateral hand numbness over five weeks. She reported that the symptoms began after a work-related incident in which a dog she was grooming suddenly pulled her forward, causing a popping sensation in her neck. She denied any associated lower extremity weakness, numbness, or falls. She also denied fever or chills, tobacco, alcohol, or illicit drug use.

An initial evaluation by her chiropractor, which included cervical spine X-rays, prompted a referral to the emergency department. At the outlying facility, a cervical spine CT revealed destructive changes at C6 and C7, prevertebral soft tissue thickening, and stranding concerning for discitis versus osteomyelitis. A CT of the head showed no acute intracranial abnormalities. She was given 50 mcg of fentanyl for pain control, started on intravenous antibiotics, and her neck was immobilized before being transferred to a tertiary care center for further evaluation and management.

On arrival at our facility, she was noted to be mildly tachycardic with a heart rate of 102 beats per minute (bpm). Other vital signs were within normal limits. Neurological examination revealed reduced strength (3/5) in the upper extremities and diminished sensation in the fifth digit bilaterally. Laboratory workup showed a normal white blood cell count (10.1 × 10⁹/L), low hemoglobin (7.8 g/dL), elevated C-reactive protein (CRP) (4.1 mg/dL), and mildly elevated procalcitonin (0.2 ng/mL).

**Figure 1:**
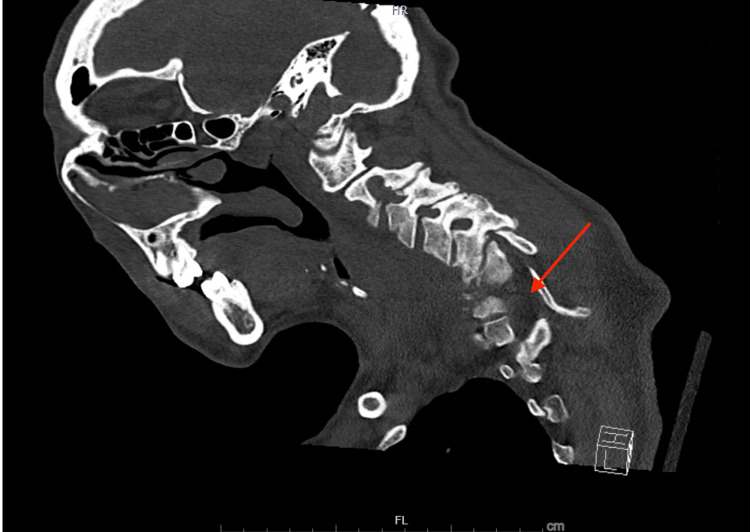
MRI of the cervical spine showing destructive changes involving C6-C7 Sagittal T2 MRI showing C6-C7 destruction with epidural phlegmon

Magnetic resonance imaging (MRI) of the cervical spine showed pronounced destructive changes from C6 to C7 (Figure 1), evidenced by significant prevertebral edema, focal kyphosis, focal disruption of the ligamentum flavum, and epidural phlegmon causing severe spinal cord compression. These findings were suggestive of cervical discitis and osteomyelitis.

The patient underwent extensive neurosurgical intervention, including anterior and posterior decompression with laminectomy, reduction, and stabilization on day one of admission, followed by C6-C7 corpectomy on hospital day three. Intraoperative findings included phlegmonous material and a small abscess. She received three units of packed red blood cells intraoperatively due to a further hemoglobin drop to 7.1 g/dL. There were no other postoperative complications.

Blood cultures and bone biopsy specimens were negative, likely due to prior initiation of intravenous antibiotics. Empiric antimicrobial therapy with Ertapenem and Daptomycin was continued for six weeks based on clinical, radiographic, and surgical findings, as recommended by the infectious disease service. The antibiotic choice was for empiric coverage for methicillin-resistant *Staphylococcus aureus* (MRSA) and Gram-negative organisms, taking into consideration the patient's history of anaphylaxis to penicillins, cephalosporins, and vancomycin.

Following surgery, the patient experienced significant neurological improvement, evidenced by strength improvement from 3/5 to 5/5 and resolution of numbness. CRP downtrended from 4.1mg/dL to 2.2mg/dL. She remained hospitalized until optimal pain control was achieved. She was subsequently discharged with plans to follow up at the infectious disease clinic two weeks post-discharge.

## Discussion

Although cervical osteomyelitis accounts for only 3-6% of all vertebral osteomyelitis cases, its proximity to the spinal cord renders it particularly dangerous, with the potential for complications such as spinal instability, neurological deficits, and systemic infection [2,4]. Early recognition and prompt management are therefore crucial to prevent irreversible neurologic damage.

While hematogenous spread remains the most common route of vertebral osteomyelitis, several risk factors have been associated with its development, including diabetes mellitus, intravenous drug use, immunosuppression, distant infectious sources, and recent trauma [5,6]. The term “post-traumatic osteomyelitis” typically refers to bone infections following open fractures or surgical interventions of closed fractures. The pathophysiology of traumatic osteomyelitis varies greatly depending on the bones involved, characteristics of the initial injury, and patient conditions. Infection may directly affect soft tissue, cortical bone, and bone marrow around the injury site. When the bone tissue is involved, the bacteria proliferate and induce an acute inflammatory reaction, resulting in necrosis of the entrapped bone [7].

Our case shares notable similarities with the case reported by Fang et al. in which a young, otherwise healthy fireman developed cervical osteomyelitis and spinal epidural abscess following minor neck trauma during a training exercise [8]. Similarly, our patient, a middle-aged dog groomer, developed cervical osteomyelitis after a seemingly trivial work-related injury, despite having no traditional predisposing factors. These cases underscore the potential for serious spinal infections even in immunocompetent individuals and highlight the importance of considering spinal osteomyelitis in the differential diagnosis of persistent neck pain post-trauma.

When diagnosed early and in the absence of neurologic deficits, cervical osteomyelitis can often be managed conservatively with intravenous antibiotics, pain control, and close monitoring. In fact, conservative treatment is successful in approximately 90% of hematogenous osteomyelitis cases without neurologic compromise [5]. The primary goals of treatment include eradication of infection, preservation of spinal function, symptom relief, and prevention of complications. In this case, however, the patient developed progressive neurologic symptoms and imaging findings consistent with spinal cord compression, necessitating prompt surgical intervention in addition to antimicrobial therapy.

Both cases also emphasize the diagnostic challenges of cervical spine infections in patients without classical risk factors. Initial imaging was inconclusive, and symptoms were nonspecific, which contributed to delayed diagnosis. As such, a high index of suspicion and close clinical follow-up are essential, particularly when symptoms persist or evolve.

## Conclusions

This case emphasizes how cervical osteomyelitis can present atypically in otherwise healthy individuals, leading to delayed diagnosis and increased risk of neurological compromise. Post-traumatic osteomyelitis should be considered even with minor trauma without open fractures.

Key takeaways include the need to maintain a broad differential when evaluating persistent neck pain, the critical role of early imaging in cases of persistent or worsening neck pain, and the necessity of initiating empiric treatment even when cultures are negative, especially after antibiotics have been administered. Raising awareness of such presentations can aid in earlier recognition, timely intervention, and improved patient outcomes in similar low-risk populations. Our findings underscore the need for future studies to better characterize the incidence and mechanisms of occult cervical infections after seemingly minor trauma.

## References

[1] Lew DP, Waldvogel FA (2004). Osteomyelitis. Lancet.

[2] Schimmer RC, Jeanneret C, Nunley PD, Jeanneret B (2002). Osteomyelitis of the cervical spine: a potentially dramatic disease. J Spinal Disord Tech.

[3] Momodu II, Savaliya V (2025). Osteomyelitis. In: StatPearls.

[4] Tsantes AG, Papadopoulos DV, Vrioni G (2020). Spinal infections: an update. Microorganisms.

[5] Carek PJ, Dickerson LM, Sack JL (2001). Diagnosis and management of osteomyelitis. Am Fam Physician.

[6] Mann S, Schütze M, Sola S, Piek J (2004). Nonspecific pyogenic spondylodiscitis: clinical manifestations, surgical treatment, and outcome in 24 patients. Neurosurg Focus.

[7] Birt MC, Anderson DW, Bruce Toby E, Wang J (2017). Osteomyelitis: recent advances in pathophysiology and therapeutic strategies. J Orthop.

[8] Fang W, Chen S, Huang D, Huang K (72). Post-traumatic osteomyelitis with spinal epidural abscess of cervical spine in a young man with no predisposing factor. J Chin Med Assoc.

